# The effect of facial attractiveness on micro-expression recognition

**DOI:** 10.3389/fpsyg.2022.959124

**Published:** 2022-09-16

**Authors:** Qiongsi Lin, Zizhao Dong, Qiuqiang Zheng, Su-Jing Wang

**Affiliations:** ^1^Key Laboratory of Behavioral Science, Institute of Psychology, Chinese Academy of Sciences, Beijing, China; ^2^Department of Psychology, University of the Chinese Academy of Sciences, Beijing, China; ^3^Teacher Education Curriculum Center, School of Educational Science, Huizhou University, Huizhou, China

**Keywords:** facial attractiveness, micro-expression, micro-expression recognition, emotion recognition, happy-face-advantage

## Abstract

Micro-expression (ME) is an extremely quick and uncontrollable facial movement that lasts for 40–200 ms and reveals thoughts and feelings that an individual attempts to cover up. Though much more difficult to detect and recognize, ME recognition is similar to macro-expression recognition in that it is influenced by facial features. Previous studies suggested that facial attractiveness could influence facial expression recognition processing. However, it remains unclear whether facial attractiveness could also influence ME recognition. Addressing this issue, this study tested 38 participants with two ME recognition tasks in a static condition or dynamically. Three different MEs (positive, neutral, and negative) at two attractiveness levels (attractive, unattractive). The results showed that participants recognized MEs on attractive faces much quicker than on unattractive ones, and there was a significant interaction between ME and facial attractiveness. Furthermore, attractive happy faces were recognized faster in both the static and the dynamic conditions, highlighting the happiness superiority effect. Therefore, our results provided the first evidence that facial attractiveness could influence ME recognition in a static condition or dynamically.

## 1. Introduction

Micro-expression (ME) is an instinctive facial movement that expresses emotion and cognition. It is difficult for individuals to identify MEs since they are rapid (usually lasting for 40–200 ms), local, low-intensity facial responses (Liang et al., 2013). On the contrary, macro-expression is easily identifiable and lasts between 500 ms and 4 s (Takalkar et al., 2021). Ekman and Friesen (1969) indicated that the only difference between ME and macro-expression is their duration. According to Shen et al. (2012), the duration of the expressions influences the accuracy of ME recognition, the proper upper limit of duration of ME may be 200 ms or less. Shen et al. (2016) utilized electroencephalogram (EEG) and event-related potentials (ERPs) and found that the EEG/ERPs neural mechanisms for recognizing MEs differ from those for recognizing macro-expressions. From their findings, the vertex positive potential (VPP) at the electrodes Cz and CPz were significantly different between MEs (duration of less than 200 ms) and macro-expressions (duration of greater than 200 ms), and the VPP amplitude of negative expression was larger than that of positive and neutral expression with the duration of less than 200 ms, while when the duration was greater than 200 ms, there was no difference in VPP amplitude induced by different emotional expressions.Previous studies discovered that emotional contexts influence ME processing at an early stage. Zhang et al. (2018) found that early ERP differences in emotional contexts on ME processing, more positive P1 (an early component related to the visual processing of faces, peaking at approximately 100 ms) and N170 (peaking at around 160 ms) elicited by targeting ME followed negative and positive contexts rather than neutral contexts. Previous functional magnetic resonance imaging (fMRI) research found that emotional contexts reduce the accuracy of ME recognition while increasing context-related activation in some emotional and attentional regions (Zhang et al., 2020). Due to the additional monitoring and attention required for emotional context inhibition, the increased perceptual load of negative and positive contexts results in increased brain activation as well as decreased behavioral performance (Siciliano et al., 2017). Studies of emotion perception have demonstrated that ME recognition is similar to macro-expression recognition and that it is affected by variety of factors, such as gender (Abbruzzese et al., 2019), age (Abbruzzese et al., 2019), occupation (Hurley, 2012), culture (Iria et al., 2019), and individual psychological characteristics (Zhang et al., 2017). ME recognition is widely used in the fields of national security, judicial interrogation, and clinical fields as an effective clue for detecting deceptions (Ekman, 2009), as MEs occurred too quickly and are very difficult to detect, scholars have long endeavored to explore and improve individuals' ability to recognize MEs. Previous studies have typically focused on how facial attractiveness moderates macro-expression recognition. To the best of our knowledge, no previous study on macro-expressions has employed facial expressions of 200 ms or less as their stimuli, it remains unclear whether the durations of facial expressions are able to modulate the effects of facial attractiveness on facial emotion recognition (FER).

Facial attractiveness is the extent to which a face makes an individual feel good and happy, and how much it makes them want to get closer to it (Rhodes, 2006). Attractiveness is a strong signal of social interaction, reflecting all facial features (Rhodes, 2006; Li et al., 2019). Attractive faces are commonly connected with good features such as personal attributes (Eagly et al., 1991; Lindeberg et al., 2019) and higher intelligence levels (Jackson et al., 1995; Mertens et al., 2021). Abundant evidence showed that facial attractiveness affects the ability to recognize facial expressions (e.g., Dion et al., 1972; Cunningham, 1986; Otta et al., 1996; Hugenberg and Sczesny, 2006; Krumhuber et al., 2007; Zhang et al., 2016). For example, Lindeberg et al. (2019) asked participants to recognize happy or angry expressions and rate the level of attractiveness of their faces, the results show that attractiveness has a strong influence on emotion perception. According to Lindeberg et al. (2019) facial attractiveness moderates expressions recognition, participants showed the happiness superiority effect for the faces with higher attractiveness levels but not for the unattractive ones, i.e., people tend to recognize happiness faster in attractive faces than in unattractive faces, while there is no such effect in other emotions recognition (i.e., anger, sadness, surprise, Leppänen and Hietanen, 2004). Li et al. (2019) also observed that facial attractiveness moderates the happiness superiority effect, participants could identify the happy expression faster in higher attractive faces, which is consistent with the findings of Lindeberg et al. (2019). Furthermore, in the study by Golle et al. (2014), the authors utilized two-alternative-forced choice paradigms, which required participants to choose one stimulus above the other. The result revealed that facial attractiveness affects happy expression recognition. When happy faces were likewise more attractive, identifying them was easier. Mertens et al. (2021) employ the mood-of-the-crowd task to compare attractive and unattractive crowds. According to the research, participants were more quick and accurate when rating happy crowds. Attractive crowds were perceived as happier than unattractive crowds, that is, people in crowds with unattractive faces were regarded to be in a negative mood, which supports the assumption that attractiveness could moderate emotion perception.

However, a few studies failed to demonstrate that facial attractiveness influences facial emotion recognition (e.g., Jaensch et al., 2014). For example, Taylor and Bryant (2016) asked participants to classify happiness, neutral, or anger emotions at two attractiveness levels (attractive, unattractive), according to the findings of their study, the detection of happiness or anger is not significantly influenced by facial attractiveness. It should be noted that Taylor and Bryant (2016) used anger as the negative expression, however, anger is often mistaken for those other emotions (Taylor and Jose, 2014), which may have contributed to the masculinization of attractive female faces that made them seem less attractive (Jaensch et al., 2014) and lead to unreliable results. Thus, this study used disgust expression as experimental material which extends the existing research. Furthermore, previous research on recognizing facial expressions has employed static stimuli, while human faces in real life are not static. As humans utilize dynamic facial expressions in everyday conversation, the ability to accurately recognize dynamic expressions makes more sense (Li et al., 2019). In contrast to static facial expressions, previous studies show that dynamic facial expressions are more ecologically valid and could induce more obvious behavioral responses, such as emotion perception (Recio et al., 2011), emotion elicitation (Scherer et al., 2019), and imitation of facial expressions (Sato and Yoshikawa, 2007). This evidence suggests that dynamic stimuli are better identified than static ones, according to face processing literature (Zhang et al., 2015). In this study, we showed participants static and dynamic stimuli to recognize MEs.

To this end, we aimed to explore whether facial attractiveness moderates ME recognition processing. In Experiment 1, static expressions of disgust, neutral, and happiness were presented. Furthermore, Experiment 2 replicated and extended Experiment 1's results by using dynamic stimuli (happy, disgust). We hypothesized that attractive faces could be judged faster overall in a static condition or dynamically; participants could recognize happiness more accurately in attractive faces than in unattractive faces.

## 2. Experiment 1

We adopted a recognition task modified from the Brief Affect Recognition Test (BART) to simulate a ME (Shen et al., 2012). In the BART paradigm (Ekman and Friesen, 1974), one of the six emotions (happiness, disgust, anger, fear, surprise, and sadness) was presented for 10 ms to 250 ms. In Experiment 1 we presented static stimuli with a duration of 200 ms (happiness as positive ME, disgust as negative ME, and neutral as a control condition) to investigate the effects of facial attractiveness on the processing of MEs. We hypothesized that participants could judge attractive faces faster in static faces, and facial attractiveness moderates the happiness superiority effect, participants could identify the happy expression faster in higher attractive faces but not for the unattractive ones.

### 2.1. Methods

#### 2.1.1. Participants

The number of participants was similar to or larger than previous research examining the effect of facial attractiveness on expression recognition (e.g., Taylor and Bryant, 2016; Li et al., 2019). Based on a *post hoc* power analysis by using G*Power 3.1 (Faul et al., 2007) and calculating power analysis for the main effect of ME (a partial η^2^ equal to 0.349, an alpha of 0.05, and a total sample size of 38) and attractiveness (a partial η^2^ equal to 0.535, an alpha of 0.05, and a total sample size of 38), we observed that this sample size generated a high power of 1-β equal to 0.978 and 0.999 separately. Thus, thirty-eight right-handed participants from Beijing Normal University, Zhuhai (*M* = 20.24 years, *SD* = 0.675 years, 20 women) were recruited and received remuneration for completing the experiment. All participants had a normal or corrected-to-normal vision and no psychiatric history. This study adhered to the Declaration of Helsinki and was approved by the Institutional Review Board of the Institute of Psychology, Chinese Academy of Sciences.

#### 2.1.2. Design

Experiment 1 adopted a 3 (ME: happy, neutral, disgust) × 2 (Attractiveness: attractive, unattractive) within-subject factors design. The dependent variables were the participants' mean accuracy score (%) and the mean reaction times (ms) for participants to accurately detect MEs.

#### 2.1.3. Materials

The Extended Cohn-Kanade Dataset (CK+) face database was used to choose images of faces (Lucey et al., 2010). CK+ is the most frequently used laboratory-controlled facial expression classification database that conforms to the Facial Action Coding System (Ekman and Friesen, 1978). At the individual (within-culture) level, Matsumoto et al. (2007) observed consistent and dependable positive connections among the response systems across all seven emotions (happiness, disgust, sadness, contempt, fear, anger, and surprise). These associations indicated that the response systems were coherent with one another. According to Ekman (1992), the response systems for anger, fear, happiness, sadness, and disgust are coherent across cultures which are based not only on a high level of agreement in the labeling of what these expressions signal across literate and preliterate cultures, but also on studies of the actual expression of emotions, both deliberately and spontaneously, as well as the association of expressions with social interactive contexts. Therefore, Caucasian faces can be used to measure Chinese college students (Zhang et al., 2017). From the CK+ face database, we picked 120 pictures of 40 different models whose facial expressions included disgust, happiness, and neutral. Twenty-two additional Chinese participants rated each neutral expression's level of attractiveness on a 7-point Likert scale (1 = very unattractive, 7 = very attractive). A paired sample *t*-test confirmed that the attractive faces (*M* = 4.18, *SD* = 0.152) were significantly higher than unattractive faces (*M* = 2.23, *SD* = 0.148), *t*_(4)_ = 15.764, *p* < 0.001. The five faces with the highest and lowest average attractiveness ratings were chosen for the research, resulting in a total of 60 trials. In these trials, ten different model faces were used for each emotion: five attractive models representing the three emotions (happiness, neutral, and disgust) and five unattractive models expressing the same emotions. All photos were 350 × 418 pixels in size and shown on a white background. A Lenovo computer (23.8-inch CRT monitor, resolution 1,920 × 1,080 pixels) and E-Prime (version 2.0) were used to present the stimuli and collect the data.

#### 2.1.4. Procedure

**Figure 1:**
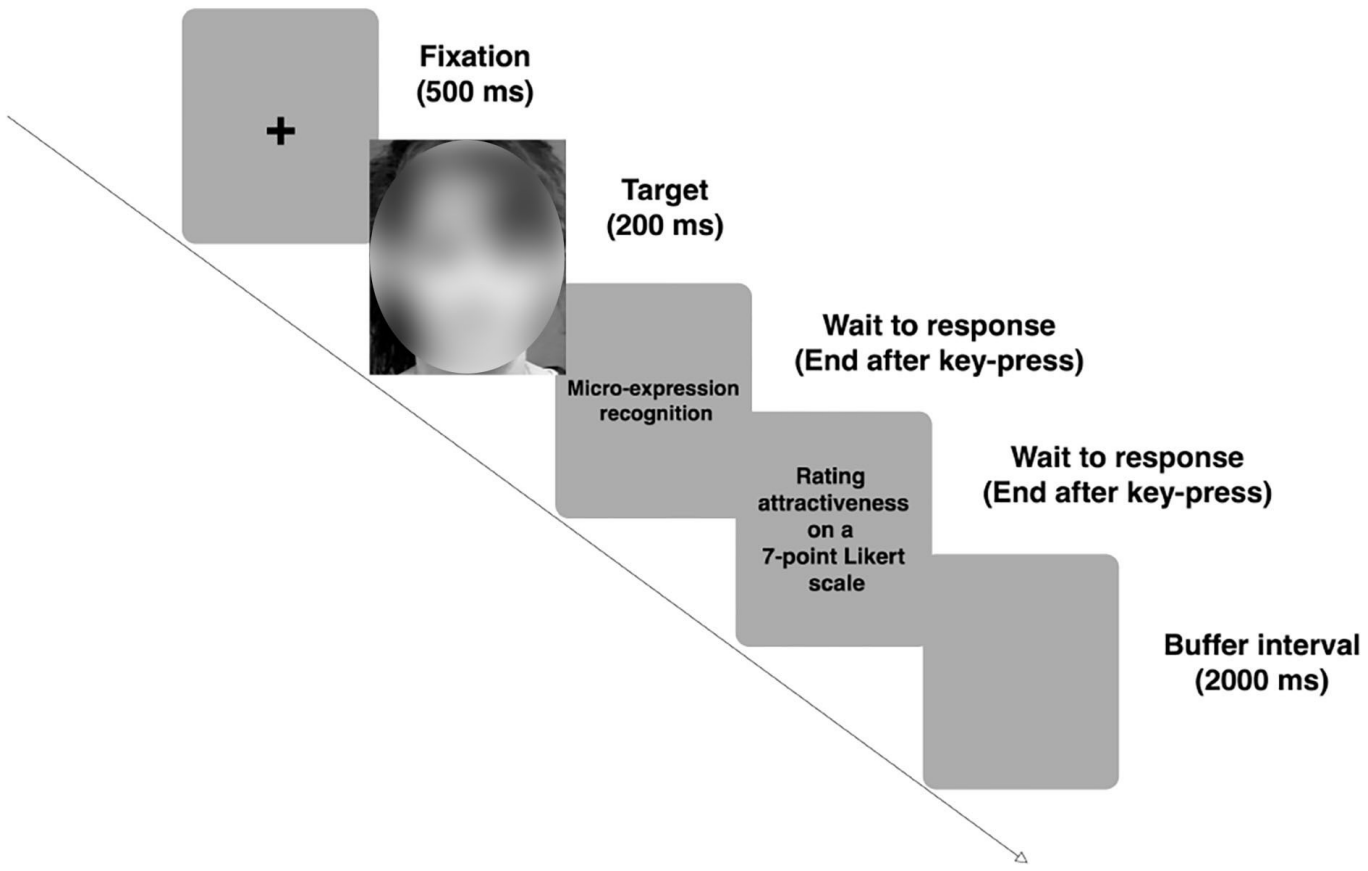
The procedure of the micro-expression recognition task and 7-point Likert rating task.

In a quiet environment, participants were tested individually. First, they were given a practice block consisting of nine trials, to begin with, so that they could get familiar with the task. It was requested of the participants that they maintain their gaze on a center fixation cross that was shown on the screen for a duration of 500 ms, then one of the three basic expressions was shown for the duration of 200 ms in the middle of the screen. Participants were told to press the appropriate key according to the micro-expression they considered the face revealed (the “J” key for happy, “K” key for neutral, or the “L” key for disgust) and rate each face on attractiveness using a 7-point Likert scale (1 = very unattractive, 7 = very attractive), each trial only displayed a single image. After 2,000 ms, the reaction screen vanished automatically. The participants were instructed to complete the task in as little time as possible while maintaining the highest level of accuracy. The experimental blocks didn't utilize the practice block's images. Each experimental block included all 30 photographs, one of each face shown twice in random order. Testing took about 15 min (refer to Figure 1).

### 2.2. Data processing

The average accuracy and mean reaction times for each combination were calculated in both experiments. To deal with the reaction time outliers, we adopted an approach suggested in Ratcliff (1993) and set up a cut-off point of 1.5 SDs above the mean. After that, the reaction time was processed in the same way as the accuracy. We utilized Greenhouse- Geisser correction for heterogeneity of covariances (if sphericity could not be assumed) and Bonferroni correction for *post-hoc* pairwise comparisons. SPSS 26.0 program was used for the data analysis.

### 2.3. Results and discussion

**Figure 2:**
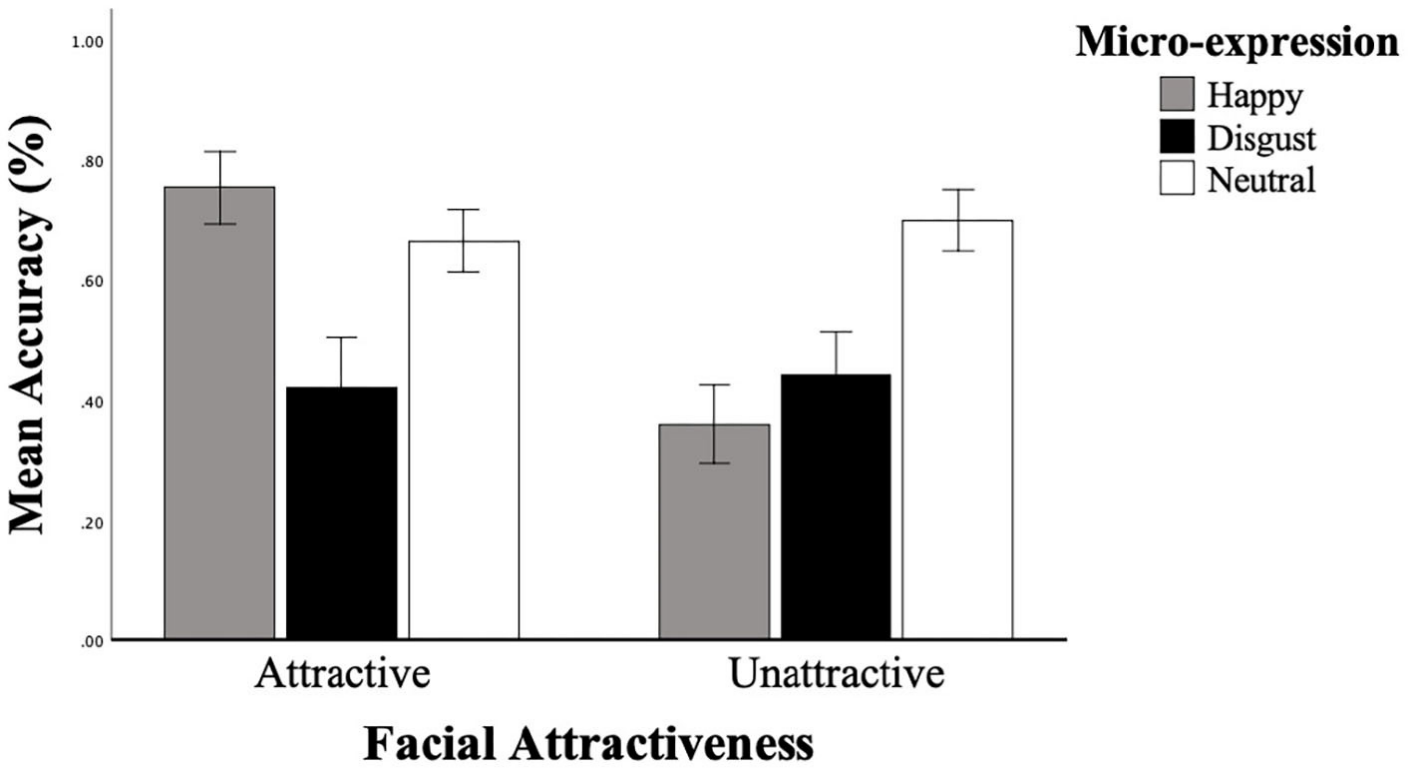
Participants' mean accuracy of the static micro-expression recognition task in two facial attractiveness levels (attractive, unattractive). Error bars reflect the 95% CIs for the mean accuracy.

We launched a 3 × 2 repeated measures ANOVA with ME (happy, neutral, disgust) and Attractiveness (attractive, unattractive) as within-subject factors, and with mean accuracy as dependent variables. The mean accuracy of the three MEs is shown in Figure 2. The results revealed a significant main effect of ME, [*F*_(2, 74)_ = 19.823, *p* < 0.001,$\eta_{p}^{2}$ = 0.349], a significant main effect of attractiveness, [*F*_(1, 37)_ = 42.519, *p* < 0.001, $\eta_{p}^{2}$ = 0.535]. The interactions between ME and attractiveness were significant, [*F*_(1.580, 2.019)_ = 41.447, *p* < 0.001, $\eta_{p}^{2}$ = 0.528]. Pairwise comparisons with Bonferroni correction show that for ME, mean accuracy were significantly higher when responding to happiness compared to disgust (*p* = 0.011, 95% *CI* [0.024, 0.228]) neutral identified higher recognition accuracy than happiness (*p* = 0.002, 95% *CI* [0.041, 0.209]), and disgust (*p* < 0.001, 95% *CI* [0.139, 0.364]). A simple main effect of ME was analyzed to examine the interaction between attractiveness and ME. The results revealed a significant simple main effect of ME under the attractive faces condition, [*F*_(2, 36)_ = 27.777, *p* < 0.001, $\eta_{p}^{2}$ = 0.607], and a significant simple main effect of ME under the unattractive faces condition, [*F*_(2, 36)_ = 38.731, *p* < 0.001, $\eta_{p}^{2}$ = 0.683]. Under the attractive faces condition, happiness (*M* = 0.755, *SD* = 0.030) identified higher recognition accuracy than disgust [*M* = 0.666, *SD* = 0.026, *t*_(36)_ = 2.34, *p* = 0.023, *d* = 0.780, 95% *CI* [0.013, 0.166]], and neutral [*M* = 0.442, *SD* = 0.036, *t*_(36)_ = 7.45, *p* < 0.001, *d* = 2.48, 95% *CI* [0.229, 0.397]], disgust identified higher recognition accuracy than neutral [*t*_(36)_ = 4.571, *p* < 0.001, *d* = 1.524, 95% *CI* [0.125, 0.322]]. Furthermore, neutral (*M* = 0.700, *SD* = 0.025) identified higher recognition accuracy than happiness [*M* = 0.421, *SD* = 0.042, *t*_(36)_ = 5.167, *p* < 0.001, *d* = 1.722, 95% *CI* [0.169, 0.389]] and disgust [*M* = 0.361, *SD* = 0.032, *t*_(36)_ = 8.692, *p* < 0.001, *d* = 2.897, 95% *CI* [0.261, 0.418]] under the unattractive faces condition, but no significant differences between happiness and disgust (*p* = 0.242, 95% *CI* [−0.043, 0.164]) (refer to Table 1).

**Table 1:** Mean accuracy of recognition of each Micro-expression in Experience 1.

	**Accuracy of recognition (%)**	
	**Attractive**	**Unattractive**
**Micro-expression**	**M ±SD**	**M ±SD**
Happy	0.775 ± 0.184	0.361 ± 0.199
Disgust	0.421 ± 0.259	0.442 ± 0.223
Neutral	0.665 ± 0.159	0.700 ± 0.156

Mean reaction times were submitted to a second repeated measures ANOVA with the same factors described above, outliers (reaction times exceeding the mean of each participant by 1.5 SD) were not included in the analysis. There was no significant main effect of ME, [*F*_(2, 56)_ = 1.661, *p* = 0.199], and attractiveness, [*F*_(1, 28)_ = 0.453, *p* = 0.507], no significant interactions between ME and attractiveness, [*F*_(2, 56)_ = 1.363, *p* = 0.264].

Attractiveness ratings were submitted to a third repeated measures ANOVA with the same factors described above. The results revealed a significant main effect of ME, [*F*_(2, 74)_ = 62.595, *p* < 0.001, $\eta_{p}^{2}$ = 0.628], a significant main effect of attractiveness, [*F*_(1, 37)_ = 64.526, *p* < 0.001, $\eta_{p}^{2}$ = 0.636]. The interactions between ME and attractiveness were significant, [*F*_(2, 74)_ = 7.786, *p* = 0.001, $\eta_{p}^{2}$ = 0.174], indicating that the attractive manipulation of the stimuli used in the current study is effective. Pairwise comparisons with Bonferroni correction show that for ME, the score of attractiveness ratings was significantly higher when responding to happiness compared to disgust (*p* < 0.001, 95% *CI* [0.500, 0.939]), and neutral (*p* < 0.001, 95% *CI* [0.427, 0.737]), neutral were rated as more attractive than disgust (*p* = 0.027, 95% *CI* [0.013, 0.264]). Further analysis revealed a significant simple main effect of ME under the attractive faces condition, [*F*_(2, 36)_ = 30.378, *p* < 0.001, $\eta_{p}^{2}$ = 0.628], and a significant simple main effect of ME under the unattractive faces condition, [*F*_(2, 36)_ = 23.264, *p* < 0.001, $\eta_{p}^{2}$ = 0.564]. Under the attractive faces condition, happiness (*M* = 4.337, *SD* = 0.164) were rated with a higher score than disgust [*M* = 3.421, *SD* = 0.135, *t*_(36)_ = 7.508, *p* < 0.001, *d* = 2.503, 95% *CI* [0.668, 1.164]], and neutral [*M* = 3.582, *SD* = 0.123, *t*_(36)_ = 7.704, *p* < 0.001, *d* = 2.568, 95% *CI* [556, 0.954]], disgust were rated with lower score than neutral [*t*_(36)_ = 2.439, *p* = 0.020, *d* = 0.813, 95% *CI* [−0.294, −0.027]]. Under the unattractive faces condition, happiness (*M* = 3.361, *SD* = 0.163) were rated with higher score than disgust [*M* = 2.837, *SD* = 0.143, *t*_(36)_ = 6.39, *p* < 0.001, *d* = 2.13, 95% *CI* [0.358, 0.690]] and neutral [*M* = 2.953, *SD* = 0.163, *t*_(36)_ = 5.826, *p* < 0.001, *d* = 1.942, 95% *CI* [0.266, 0.550]], no significant differences between disgust and neutral [*t*_(36)_ = 1.634, *p* = 0.112, *d* = 0.545, 95% *CI* [−0.260, 0.029]].

In this study, we examine how facial attractiveness influences the processing of ME recognition in static conditions. Analysis of accuracy indicated that the recognition of ME is influenced by attractiveness. Participants categorized attractive faces more accurately than unattractive faces. Specifically, participants showed the happiness superiority effect for the faces with higher attractiveness levels but not for the unattractive ones, the expression of happiness on the attractive faces was the easiest to recognize, followed by neutral, and then disgust.

## 3. Experiment 2

In Experiment 2, we presented dynamic stimuli to investigate the effects of facial attractiveness on the processing of MEs. We hypothesized that participants could judge attractive faces faster overall in a dynamic context; participants showed the happiness superiority effect for the faces with higher attractiveness levels but not for the unattractive ones.

### 3.1. Methods

Experiment 2 employed a 2 (ME: happy, disgust) × 2 (Attractiveness: attractive, unattractive) within-subject factors design. The dependent variables were the participants' mean accuracy score (%) and the mean reaction times (ms) for participants to accurately detect MEs. Participants and procedure were the same as in Experiment 1. Based on a *post-hoc* power analysis by using G*Power 3.1 (Faul et al., 2007) and calculating power analysis for the main effect of attractiveness (a partial η^2^ equal to 0.436, an alpha of 0.05, and a total sample size of 38), we observed that this sample size generated a high power of 1-β equal to 0.999. To exclude practice effects, we balanced the order of Experiment 1 and Experiment 2 between participants. Thirty-eight participants were randomly divided into two groups (Group A and B), each comprised of 19 participants. Group A completed Experiment 1 follow by Experiment 2, and Group B did the opposite. Also, we used the materials from Experiment 1 to create short video clips. Shen et al. (2012) found a significant difference in recognition accuracy with durations of 40 ms and 120 ms under the METT paradigm condition; however, when the duration was greater than 120 ms, there was no difference in accuracy rate. Thus, we employ the intermediate values with a duration of 80 ms as the target stimulus. Based on the neutral-emotional-neutral paradigm (Zhang et al., 2014), we used neutral as the context expression in this experiment. Zhang et al. (2014) indicated that MEs are contained in the flow of expressions including both neutral and other emotional MEs, considering that a ME is occurred very fast and is always submerged in other MEs, the neutral faces before and after the target ME were presented for 60 ms in order to simulate the real situation in which the ME happened, with happiness or disgust flashed briefly for 80 ms, resulting in a total of 200 ms. Thus, the dynamic stimuli consisted of 20 clips (each clip lasting for 200 ms and showing the same model), comprised of two levels of Attractiveness (attractive and unattractive) and presented as two stimulus types (neutral-happiness-neutral and neutral-disgust-neutral) for each of the 10 models, each clip was shown twice in random order. E-Prime (version 3.0) was used to show the stimuli and collect the data.

### 3.2. Results and discussion

**Figure 3:**
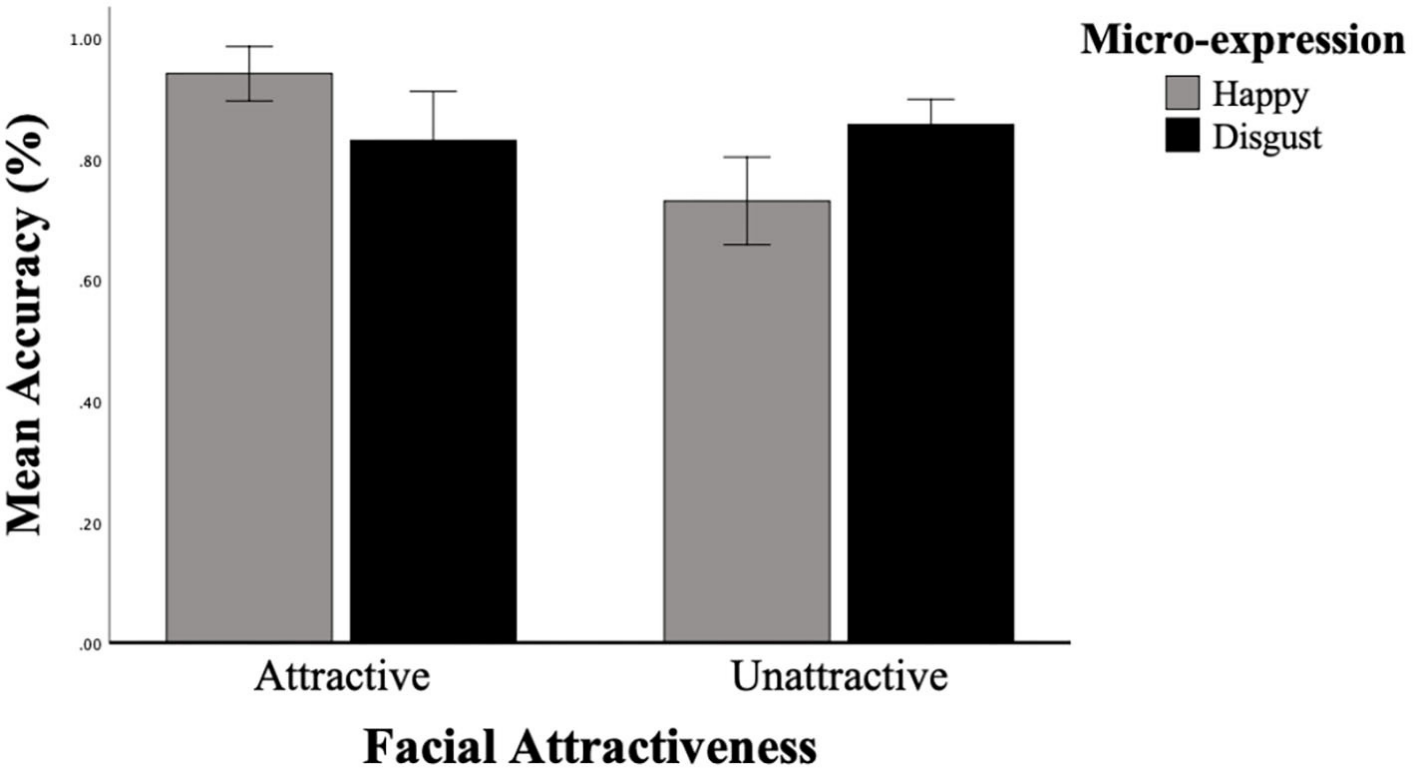
Participants' mean accuracy of the dynamic micro-expression recognition task in two facial attractiveness levels (attractive, unattractive). Error bars reflect the 95% CIs for the mean accuracy.

We launched a 2 × 2 repeated measures ANOVA with ME (happy, disgust) and Attractiveness (attractive, unattractive) as within-subject factors, and with mean accuracy as dependent variables. The mean accuracy of the two MEs is shown in Figure 3. The results revealed a significant main effect of attractiveness, [*F*_(1, 37)_ = 28.560, *p* < 0.001, $\eta_{p}^{2}$ = 0.436]. The main effect of ME was not significant, [*F*_(1, 37)_ = 0.062, *p* = 0.805]. The interactions between ME and attractiveness were significant, [*F*_(1, 37)_ = 14.637, *p* < 0.001, $\eta_{p}^{2}$ = 0.283]. A simple main effect of ME was analyzed to examine the interaction between attractiveness and ME. The results revealed a significant simple main effect of ME under the attractive faces condition, [*F*_(1, 37)_ = 5.512, *p* = 0.024, $\eta_{p}^{2}$ = 0.130], and a significant simple main effect of ME under the unattractive faces condition, [*F*_(1, 37)_ = 9.294, *p* = 0.004, $\eta_{p}^{2}$ = 0.201]. Furthermore, happiness (*M* = 0.942, *SD* = 0.022) identified higher recognition accuracy than disgust [*M* = 0.732, *SD* = 0.036, *t*_(37)_ = 2.362, *p* = 0.024, *d* = 0.777, 95% *CI* [0.015, 0.206]] under the attractive faces condition, happiness (*M* = 0.832, *SD* = 0.040) identified lower recognition accuracy than disgust [*M* = 0.858, *SD* = 0.021, *t*_(37)_ = 3.073, *p* = 0.004, *d* = 1.010, 95% *CI* [−0.210, −0.042]] under the unattractive faces condition (refer to Table 2).

**Table 2:** Mean accuracy of recognition of each Micro-expression in Experience 2.

	**Accuracy of recognition (%)**	
	**Attractive**	**Unattractive**
**Micro-expression**	**M ±SD**	**M ±SD**
Happy	0.942 ± 0.136	0.731 ± 0.221
Disgust	0.731 ± 0.221	0.857 ± 0.127

Mean reaction times were submitted to a second repeated measures ANOVA with the same factors described above, outliers (reaction times exceeding the mean of each participant by 1.5 SD) were not included in the analysis. There was no significant main effect of ME, [*F*_(1, 35)_ = 0.218, *p* = 0.644], or a significant main effect of attractiveness, [*F*_(1, 35)_ =2.492, *p* = 0.123]. Remarkably, the interaction of ME × Attractiveness was significant, [*F*_(1, 35)_ = 21.245, *p* < 0.001, $\eta_{p}^{2}$ = 0.378]. A follow-up simple effect analysis was employed to investigate the effect of ME within each level of attractiveness. The results revealed a significant simple main effect of ME under the attractive faces condition, [*F*_(1, 37)_ = 9.267, *p* = 0.004, $\eta_{p}^{2}$ = 0.200], and a significant simple main effect of ME under the unattractive faces condition, [*F*_(1, 37)_ = 21.773, *p* < 0.001, $\eta_{p}^{2}$ = 0.370]. Happiness (*M* = 758.280, *SD* = 55.873) identified faster than disgust (*M* = 919.013, *SD* = 79.390) under the attractive faces condition [*t*_(37)_ = 3.044, *p* = 0.004, *d* = 1.001, 95% *CI* [–267.715, –53.752]], disgust (*M* = 821.605, *SD* = 66.602) identified faster than happiness (*M* = 982.400, *SD* = 76.192) under the unattractive faces condition [*t*_(37)_ = 4.666, *p* < 0.001, *d* = 1.534, 95% *CI* [–230.616, –90.973]].

Attractiveness ratings were submitted to a third repeated measures ANOVA with the same factors described above. The results revealed a significant main effect of ME, [*F*_(1, 37)_ = 62.947, *p* < 0.001, $\eta_{p}^{2}$ = 0.630], a significant main effect of attractiveness, [*F*_(1, 37)_ = 101.369, *p* < 0.001, $\eta_{p}^{2}$ = 0.733]. The interactions between ME and attractiveness were significant, [*F*_(1, 37)_ = 20.428, *p* < 0.001, $\eta_{p}^{2}$ = 0.356], indicating that the attractive manipulation of the stimuli used in the current study is effective. Further analysis revealed a significant simple main effect of ME under the attractive faces condition, [*F*_(1, 37)_ = 143.607, *p* < 0.001, $\eta_{p}^{2}$ = 0.795], and a significant simple main effect of ME under the unattractive faces condition, [*F*_(1, 37)_ = 29.711, *p* < 0.001, $\eta_{p}^{2}$ = 0.445]. Under the attractive faces condition, happiness (*M* = 4.471, *SD* = 0.173) was rated with a higher score than disgust [*M* = 3.195, *SD* = 0.167, *t*_(37)_ = 11.925, *p* < 0.001, *d* = 3.921, 95% *CI* [1.061, 1.492]]. Under the unattractive faces condition, happiness (*M* = 3.374, *SD* = 0.132) was rated with a higher score than disgust [*M* = 2.682, *SD* = 0.146, *t*_(37)_ = 5.449, *p* < 0.001, *d* = 1.792, 95% *CI* [0.435, 0.949]].

In this study, we examine how facial attractiveness influences the processing of ME recognition in dynamic conditions. Analysis of accuracy indicated that attractiveness affects ME recognition. Participants could recognize attractive faces more accurately. Specifically, we observed a higher accuracy rate for happiness than disgust under the attractive faces condition, which supports the assumption that attractiveness could moderate the happiness superiority effect. For the response times, the interaction of Attractiveness × ME was significant, attractive faces were recognized faster than unattractive faces, and happiness was categorized faster than disgust under the attractive face condition whereas this happiness superiority effect did not apply to unattractive faces. According to the results of attractiveness ratings, the advantage of happy faces may be caused by their attractiveness. Overall, participants could identify the happy expression faster and more accurately in higher attractive faces, demonstrating that participants have a stronger ability to identify dynamic expressions that are very attractive.

## 4. General discussion

Across two experiments, we showed participants static and dynamic faces to recognize MEs. We revealed evidence of the effect of attractiveness on the recognition of ME in either static conditions or dynamically. The results suggest that these two attributes (Attractiveness × ME) are strongly interconnected. Participants showed the happiness superiority effect for the faces with higher attractiveness levels but not for the unattractive ones in both experiments. These findings are in line with the attractiveness stereotype, which defines the phenomena in which individuals correlate physical appearance with a variety of beneficial qualities (Eagly et al. 1991). For instance, attractiveness could boost job interview chances (Watkins and Johnston, 2000). According to the attractiveness stereotype, attractive appearance and good qualities have a strong association with the thoughts of people. Therefore, the identification of attractive faces and positive emotions may be rewarded with an advantage, enhancing their speedy recognition (Golle et al., 2014).

The happiness superiority effect was strengthened by neuroimaging evidence indicating that the medial frontal cortex plays an important role in happy face recognition (Kesler et al., 2001). Ihme et al. (2013) used functional magnetic resonance imaging (fMRI) for the first time to explore the brain mechanism of JACBART and revealed increasing activation with higher performance in the basal ganglia for the negative faces and orbitofrontal areas for happiness and anger. Furthermore, previous research implicated that basal ganglia and orbitofrontal cortex are both involved in the processing of emotional facial expressions. According to O'Doherty et al. (2003), the medial orbitofrontal cortex (OFC) is a region that is known involved in representing stimulus reward value and was shown to be more active when an attractive face was associated with a happy expression, rather than a neutral one. Further studies should find out whether facial attractiveness that correlates with the detection performance of MEs predicts activation in basal ganglia and orbitofrontal cortex.

In general, this study aimed to explore the effects of facial attractiveness on the processing of MEs in static and dynamic experimental conditions. The findings of our study verified and represent an extension of previous research. On one hand, the results show that participants could identify the happy expression quicker in higher attractive faces, which supports the happiness superiority effect and strengthens this theory with more evidence. On the other hand, this research suggests that the moderation of ME recognition is not limited to invariant facial attributes (such as gender and race) but also applies to variable face features such as facial attractiveness. Furthermore, previous studies suggest that ME recognition training has significant effects on the recognition of MEs (Matsumoto and Hwang, 2011). However, the selection of stimulus material in prior research may not address the variations in the attractiveness of the faces representing the various groups. The current findings demonstrate that facial attractiveness is processed quickly enough to influence ME recognition; hence, facial attractiveness should be considered when selecting faces as stimuli for ME recognition training. Also, since individuals can be trained to recognize MEs more accurately and quickly in as little as a few hours, the effects of facial attractiveness on ME recognition may be reduced when individuals receive ME training.

The present experiments entailed several limitations. First, this research only used two basic expressions as experimental materials. It remains unclear whether facial attractiveness affects other MEs (such as a sadness expression) as much as in our research, a wider range of facial expressions should be examined in future research. Second, we used synthetic MEs in the experiences, while natural MEs may be shorter, asymmetrical, and weaker than synthetic MEs, future research could use natural MEs with more ecological validity as research materials. However, this would require a ME database with a rich sample. Third, we employed the Caucasian faces as experimental materials, which were outgroup members to the participants of the current study. However, evidence from cross-cultural studies suggests that the ME recognition process might differ between the ingroup members and outgroup members. For example, Elfenbein and Ambady (2002) suggested that individuals are more accurate at identifying ingroup emotions since they are more familiar with their own race expressions and faces. Therefore, it may be useful to use a wider variety of face types in future studies to evaluate the ingroup advantage in ME recognition-related facial attractiveness in a context of stimulus equivalence. Finally, since a ME is often embedded in the flow of other MEs, we employed 80 ms for target MEs, and the neutral MEs before and after the emotional MEs were only presented for 60 ms to simulate the actual situation in which the ME occurred. This led to the neutral expressions and target ME being combined and the entire duration was examined. Future studies could employ an ERP experiment to investigate the modulation of early visual processing (e.g., P1 and N170) by using natural MEs in order to investigate the neural mechanism for the effect of facial attractiveness on ME. Moreover, this research only examined the presentation time of MEs at 200 ms. Shen et al. (2012) showed that the accuracy of MEs recognition depends on how long they last and reaches a turning point at 200 ms or maybe even less than 200 ms before leveling off. This suggests that the critical time point that differentiates MEs may be 1/5 of a second. Does facial attractiveness have different effects on ME recognition with longer and shorter presentation times? These questions need to be further explored.

## 5. Conclusion

In conclusion, the current research provides objective evidence that facial attractiveness influences the processing of MEs. Specifically, we observed that attractive happy faces can be recognized faster and more accurately, emphasizing the happiness superiority effect whether in a static condition or dynamically. Moreover, these new results support the assumption that facial attractiveness could moderate emotion perception. Further studies should employ eye tracker technology to detect visual attention mechanisms in MEs processing that is influenced by facial attractiveness.

## Data availability statement

The raw data supporting the conclusions of this article will be made available by the authors, without undue reservation.

## Ethics statement

The studies involving human participants were reviewed and approved by Institute of Psychology, Chinese Academy of Sciences, Beijing, China. The patients/participants provided their written informed consent to participate in this study.

## Author contributions

QL has contributed the main body of text and the main ideas. ZD has contributed to the construction of the text and refinement of ideas and provided extensive feedback and commentary. QZ was responsible for constructing the partial research framework. S-JW led the project and acquired the funding support. All authors contributed to the article and approved the submitted version.

## Funding

This article was supported by grants from the National Natural Science Foundation of China (U19B2032).

## Conflict of interest

The authors declare that the research was conducted in the absence of any commercial or financial relationships that could be construed as a potential conflict of interest.

## Publisher's note

All claims expressed in this article are solely those of the authors and do not necessarily represent those of their affiliated organizations, or those of the publisher, the editors and the reviewers. Any product that may be evaluated in this article, or claim that may be made by its manufacturer, is not guaranteed or endorsed by the publisher.
